# Stigmatizing Substance Use Terminology in Grant Abstracts Following High-Level Language Guidance

**DOI:** 10.1001/jamanetworkopen.2024.57762

**Published:** 2025-02-04

**Authors:** Evan L. Eschliman, Haruka Kokaze, Long Jie Huang, Karen Choe, Pia M. Mauro

**Affiliations:** 1 Department of Epidemiology, Columbia University Mailman School of Public Health, New York, New York; 2 Department of Applied Psychology, New York University, New York; 3 Department of Educational and Counseling Psychology, University at Albany, State University of New York, Albany; 4 Department of Social and Behavioral Sciences, New York University School of Global Public Health, New York

## Abstract

This cross-sectional study examines the use of stigmatizing terms in US grants funded by the National Institute on Drug Abuse following changes to official guidance on the language of substance use disorders in 2017 and 2021.

## Introduction

Stigmatizing language related to substance use has negative consequences,^1,2^ and people with lived and living experience have long advocated for the use of humanizing, nonstigmatizing language.^1,2^ High-level efforts to recast substance-related language include the shift in formal diagnostic labels in the *Diagnostic and Statistical Manual of Mental Disorders* (Fifth Edition) (*DSM-5*) to “substance use disorders” in 2013 and the issuance of guidance on nonstigmatizing language by organizations such as the White House Office of National Drug Control Policy in early 2017^3^ and the National Institute on Drug Abuse (NIDA) in early 2021.^4^ To assess how high-level language guidance may have affected researchers’ use of stigmatizing substance use terminology, we examined its use in abstracts of NIDA-funded grants and tested whether observed changes accelerated in 2017 and 2021.

## Methods

We retrieved data on all 6065 NIDA-funded grants in their first year of support from fiscal years (FYs) 2013 to 2023 using the National Institutes of Health Research Portfolio Online Reporting Tools Expenditures and Results (NIH RePORTER) tool. We identified 9 stigmatizing terms from NIDA’s Words Matter guidance^4^ specific to substance use behavior (*abuse*, *habit*) and people who use substances (*abuser*, *addict*, *addicted baby*, *alcoholic*, *drunk*, *junkie*, *user*). We systematically searched grants’ abstracts for these 9 terms; our search syntax excluded *abuse* when naming NIDA (eTable in the Supplement).

We calculated yearly percentages of grant abstracts using stigmatizing terminology (ie, by any term, category of term, and each term). We used generalized linear models with a binomial distribution and log link to estimate the relative risk of using stigmatizing terminology. We first treated FY as a linear variable (model A), then tested for associations between using stigmatizing terminology and time since the specified years of high-level language guidance using piecewise linear spline functions (knots used were FY 2017 and FY 2021) (model B). We considered associations statistically significant if the 95% CI did not include 1, conducted analyses using Stata SE version 18 (StataCorp), and followed Strengthening the Reporting of Observational Studies in Epidemiology (STROBE) reporting guidelines. This study was deemed exempt by the institutional review board.

## Results

Overall, 286 of 498 grant abstracts (53.8%) used any stigmatizing term in FY 2013, and 163 of 639 (25.5%) used any stigmatizing term in FY 2023 (Figure). The use of stigmatizing terminology for substance use behavior was consistently higher than for people who use substances. *Abuse* was most used across all years, followed by *user*.

**Figure.**
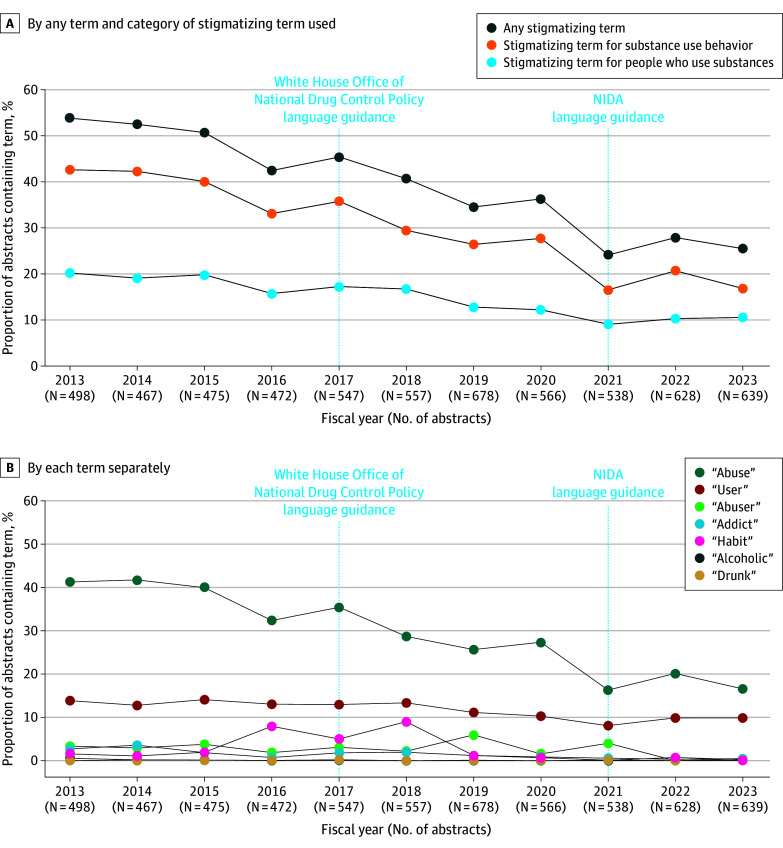
Grant Abstracts Using Stigmatizing Terminology Related to Substance Use, Fiscal Years 2013 to 2023 Grant abstracts were all abstracts of grants funded by the National Institute on Drug Abuse (NIDA) in their first fiscal year of support. Stigmatizing terms for substance use behavior include *abuse* and *habit*. Stigmatizing terms for people who use substances include *addict*, *user*, *abuser*, *alcoholic*, and *drunk*. The terms *junkie* and *addicted baby* were not used in this period and are thus not reflected in either graph.

In model A, we saw an 8% average yearly reduction in using any stigmatizing term (risk ratio [RR], 0.92; 95% CI, 0.92-0.93) (Table). In model B, we saw a faster yearly rate of change in FYs 2017 through 2021 compared with before FY 2017 (spline term RR, 0.94; 95% CI, 0.89-0.98). The differences in rates of change after FY 2021 were not statistically significantly different than before 2017 but signaled marginally slower decreases than FYs 2017 to 2021 (spline term RR, 1.08; 95% CI, 0.98-1.19). Findings were similar for both term categories.

**Table.** Generalized Linear Models of the Use of Stigmatizing Terminology Related to Substance Use in Abstracts of Grants Over Time and Following High-Level Guidance Issued in 2017 and 2021

FYs and periods corresponding to high-level language guidance	Use of stigmatizing term, RR (95% CI)		
	Any	For substance use behavior	For people who use substances
Model A			
Average yearly change per FY	0.92 (0.92-0.93)^a^	0.91 (0.90-0.92)^a^	0.92 (0.91-0.94)^a^
Model B			
Yearly change, FY 2013-2017^b^	0.95 (0.93-0.98)^a^	0.95 (0.92-0.98)^a^	0.97 (0.92-1.02)
Yearly change, FY 2017-2021^c^	0.89 (0.86-0.92)^a^	0.88 (0.84-0.91)^a^	0.87 (0.82-0.92)^a^
Yearly change, FY 2021-2023^d^	0.96 (0.89-1.04)	0.93 (0.84-1.02)	1.03 (0.89-1.18)
Difference in yearly change: FY 2017-2021 vs FY 2013-2017	0.94 (0.89-0.98)^a^	0.93 (0.87-0.99)^a^	0.90 (0.82-0.99)^a^
Difference in yearly change: FY 2021-2023 vs FY 2017-2021	1.08 (0.98-1.19)	1.06 (0.93-1.20)	1.18 (0.98-1.42)
Difference in yearly change: FY 2021-2023 vs FY 2013-2017	1.01 (0.93-1.09)	0.98 (0.89-1.08)	1.06 (0.92-1.23)

Abbreviations: FY, fiscal year; NIDA, National Institute on Drug Abuse; RR, risk ratio.

^a^ *P* < .05.

^b^ Corresponds to the period prior to White House Office of National Drug Control Policy language guidance in 2017.

^c^ Corresponds to the period after White House Office of National Drug Control Policy language guidance in 2017 and prior to NIDA language guidance in 2021.

^d^ Corresponds to the period after NIDA language guidance in 2021.

## Discussion

The use of stigmatizing terminology in NIDA-funded grant abstracts has decreased by over half since 2013. This overall decrease and the accelerated decrease after 2017 could suggest researchers were responsive to high-level language guidance and may largely recognize the harms of stigmatizing language. Still, 1 in 4 abstracts contained a stigmatizing term in 2023, and there was no accelerated decrease in stigmatizing terminology use after 2021. Further decreases will likely require additional institutional follow-through (eg, the proposed renaming of NIDA to National Institute on Drugs and Addiction) and structural and collective efforts beyond language guidance alone. Future work could address limitations of this study by investigating other high-level efforts, a broader range of terms and phrases, and other types of texts. Continuing to decrease the use of stigmatizing language is a necessary—but not sufficient—step toward ending stigma toward substance use.^5,6^

## Notes

Supplement 1. **eTable.** Search Syntax for the 9 Stigmatizing Terms Selected From NIDA’s Words Matter Guidance

Supplement 2.
Data Sharing Statement
