# Supplementary material for: Stigmatizing Substance Use Terminology in Grant Abstracts Following High-Level Language Guidance
Source: JAMA Netw Open. 2025 Feb 4;8(2):e2457762. doi: 10.1001/jamanetworkopen.2024.57762 (PMC11795323; doi:10.1001/jamanetworkopen.2024.57762)
Supplement: Supplement 1. — eTable. Search Syntax for the 9 Stigmatizing Terms Selected From NIDA’s Words Matter Guidance [file jamanetwopen-e2457762-s001.pdf]

Supplemental Online Content

Eschliman EL, Kokaze H, Huang LJ, Choe K, Mauro PM. Stigmatizing substance use terminology in grant abstracts following high-level language guidance. *JAMA Netw Open.* 2025;8(2):e2457762. doi:10.1001/jamanetworkopen.2024.57762

eTable. Search Syntax for the 9 Stigmatizing Terms Selected From NIDA's Words Matter Guidance

This supplemental material has been provided by the authors to give readers additional information about their work.

© 2025 Eschliman EL et al. *JAMA Network Open*.

eTable. Search Syntax for the 9 Stigmatizing Terms Selected From NIDA’s Words Matter Guidance

**Term**	**Search Syntax**
addict	" addict "<br>“ addict.”<br>“ addict,”<br>“ addict;”<br>" addicts "<br>“ addicts.”<br>“ addicts,”<br>“ addicts;”
user	" user "<br>" user."<br>" user,"<br>" user;"<br>" users "<br>" users."<br>" users,"<br>" users;"
junkie	" junkie "<br>“ junkie.”<br>“ junkie,”<br>“ junkie;”<br>" junkies "<br>“ junkies.”<br>“ junkies,”<br>“ junkies;”
alcoholic	" alcoholic "<br>“ alcoholic.”<br>“ alcoholic,”<br>“ alcoholic;”<br>" alcoholics "<br>“ alcoholics.”<br>“ alcoholics,”<br>“ alcoholics;”
drunk	“ drunk ”<br>“ drunk.”<br>“ drunk,”

© 2025 Eschliman EL et al. *JAMA Network Open*.

	“ drunk;”<br>“ drunks ”<br>“ drunks.”<br>“ drunks,”<br>“ drunks;”
habit	“ habit ”<br>“ habit.”<br>“ habit,”<br>“ habit;”<br>“ habits ”<br>“ habits.”<br>“ habits,”<br>“ habits;”
abuse	" abuse "<br>" abuse."<br>" abuse,"<br>" abuse;"<br>" abuses "<br>" abuses."<br>" abuses,"<br>" abuses;"<br><br>*Exclude if the term is mentioned in "National Institute on Drug Abuse"
addicted baby	" addicted baby "<br>" addicted baby. "<br>" addicted baby, "<br>" addicted baby; "
abuser	" abuser "<br>" abuser."<br>" abuser,"<br>" abuser;"<br>" abusers "<br>" abusers."<br>" abusers,"<br>" abusers;"

NIDA’s *Words Matter* guidance is available at the following link: https://nida.nih.gov/research-topics/addiction-science/words-matter-preferred-language-talking-about-addiction

© 2025 Eschliman EL et al. *JAMA Network Open*.
