# Supplementary material for: Stigmatizing Substance Use Terminology in Grant Abstracts Following High-Level Language Guidance
Source: JAMA Netw Open. 2025 Feb 4;8(2):e2457762. doi: 10.1001/jamanetworkopen.2024.57762 (PMC11795323; doi:10.1001/jamanetworkopen.2024.57762)
Supplement: Supplement 2. — Data Sharing Statement [file jamanetwopen-e2457762-s002.pdf]

Data Sharing Statement

Eschliman. Stigmatizing Substance Use Terminology in Grant Abstracts Following High-Level Language Guidance. *JAMA Netw Open*. Published February 04, 2025. doi:10.1001/jamanetworkopen.2024.57762

Data
Data available: Yes
Data types: Data (not involving human participants)
How to access data: Available upon request to corresponding author at evan.e@columbia.edu.
When available: With publication

Supporting Documents
Document types: None

Additional Information
Who can access the data: Anyone requesting the data.
Types of analyses: For any purpose.
Mechanisms of data availability: Without investigator support.
